# Neutrophil-Derived Myeloperoxidase Aggravates Non-Alcoholic Steatohepatitis in Low-Density Lipoprotein Receptor-Deficient Mice

**DOI:** 10.1371/journal.pone.0052411

**Published:** 2012-12-20

**Authors:** Sander S. Rensen, Veerle Bieghs, Sofia Xanthoulea, Evi Arfianti, Jaap A. Bakker, Ronit Shiri-Sverdlov, Marten H. Hofker, Jan Willem Greve, Wim A. Buurman

**Affiliations:** 1 Department of Surgery, NUTRIM School for Nutrition, Toxicology and Metabolism, Maastricht University Medical Centre, Maastricht, The Netherlands; 2 Department of Genetics and Cell Biology, NUTRIM School for Nutrition, Toxicology and Metabolism, Maastricht University Medical Centre, Maastricht, The Netherlands; 3 Department of Clinical Genetics, NUTRIM School for Nutrition, Toxicology and Metabolism, Maastricht University Medical Centre, Maastricht, The Netherlands; 4 Department of Pathology & Medical Biology, Medical Biology Section, Molecular Genetics, University Medical Center Groningen, University of Groningen, Groningen, The Netherlands; 5 Department of Surgery, Atrium Medical Center Parkstad, Heerlen, The Netherlands; The University of Hong Kong, Hong Kong

## Abstract

**Background:**

Chronic inflammation and oxidative stress play fundamental roles in the pathogenesis of non-alcoholic steatohepatitis (NASH). Previously, we reported that myeloperoxidase (MPO), an aggressive oxidant-generating neutrophil enzyme, is associated with NASH severity in man. We now investigated the hypothesis that MPO contributes to the development and progression of NASH.

**Methodology:**

Low-density lipoprotein receptor-deficient mice with an MPO-deficient hematopoietic system (*LDLR^−/−/^MPO^−/−tp^* mice) were generated and compared with *LDLR^−/−/^MPO^+/+tp^* mice after induction of NASH by high-fat feeding.

**Results:**

High-fat feeding caused a ∼4-fold induction of liver MPO in *LDLR^−/−/^MPO^+/+^* mice which was associated with hepatic sequestration of MPO-positive neutrophils and high levels of nitrotyrosine, a marker of MPO activity. Importantly, *LDLR^−/−/^MPO^−/−tp^* mice displayed markedly reduced hepatic neutrophil and T-lymphocyte infiltration (p<0.05), and strong down regulation of pro-inflammatory genes such as TNF-α and IL-6 (p<0.05, p<0.01) in comparison with *LDLR^−/−/^MPO^+/+tp^* mice. Next to the generalized reduction of inflammation, liver cholesterol accumulation was significantly diminished in *LDLR^−/−/^MPO^−/−tp^* mice (p = 0.01). Moreover, MPO deficiency appeared to attenuate the development of hepatic fibrosis as evident from reduced hydroxyproline levels (p<0.01). Interestingly, visceral adipose tissue inflammation was markedly reduced in *LDLR^−/−/^MPO^−/−tp^* mice, with a complete lack of macrophage crown-like structures. In conclusion, MPO deficiency attenuates the development of NASH and diminishes adipose tissue inflammation in response to a high fat diet, supporting an important role for neutrophils in the pathogenesis of metabolic disease.

## Introduction

The progression of non-alcoholic steatohepatitis (NASH) is driven by activation of the innate immune system, which contributes to hepatocyte damage and fibrosis in various ways [1]. Both Kupffer cells and the complement system have been shown to be involved [2], [3]. Furthermore, neutrophil accumulation is a prominent feature of the inflammation observed in NASH [4], [5]. These phagocytes are notorious for their ability to induce tissue damage through generation of aggressive oxidants, which is largely mediated by the myeloperoxidase (MPO) enzyme [6], [7]. Importantly, increased MPO activity has previously been suggested to promote lipid peroxidation in steatotic livers [4], a process involved in the progression of simple steatosis to steatohepatitis. Recently, we obtained additional evidence implicating MPO in the progression of NASH by showing that accumulation of HOCl-modified proteins and nitrated proteins was associated with increased hepatic CXC chemokine expression in the liver of patients with NASH [5]. MPO also catalyzes nitration of protein tyrosyl groups, which is associated with human non-alcoholic fatty liver disease (NAFLD) as well [5], [8].

Next to its ability to induce tissue damage, MPO also directly regulates inflammatory pathways and processes involved in fibrosis. For example, MPO enhances macrophage cytotoxicity [9] and induces neutrophil activation [10]. In addition, MPO-derived HOCl causes fragmentation of the extracellular matrix [11], resulting in activation of hepatic stellate cells.

All in all, there is compelling evidence to suggest that MPO plays a crucial role in the pathogenesis of NASH by affecting inflammation, oxidative stress, and fibrogenesis. We now report on studies with NASH-prone [12] low-density lipoprotein receptor-deficient mice (*LDLR* ^−/−^ mice) transplanted with MPO^−/−^ or MPO^+/+^ bone marrow. Our data demonstrate that MPO deficiency attenuates hepatic cholesterol accumulation, inflammation, and potentially fibrosis in response to a high-fat diet, indicating an important role for MPO in metabolic liver disease.

## Materials and Methods

### Ethics Statement

This study was carried out in strict accordance with the recommendations in the Guide for the Care and Use of Laboratory Animals of the National Institutes of Health. The protocol was approved by the Committee for Animal Welfare of Maastricht University (Permit Number: 2007-034). The investigation conforms to the Guide for the Care and Use of Laboratory Animals published by the US National Institutes of Health (NIH Publication No. 85-23, revised 1996).

### Mice

Twelve weeks old female *LDLR^−/−^* (Jackson Laboratory, Bar Harbor, Maine) and *MPO^−/−^* [13] or *MPO* ^+/+^ mice, on a C57BL/6J background, were randomly assigned to the *LDLR^−/−/^MPO^−/−tp^* (n = 9) or the *LDLR^−/−/^MPO* ^+/+tp^ (n = 9) group. *LDLR* ^−/−^ mice were subjected to 11 Gy of radiation. The following day, 10^7^ bone marrow cells of *MPO^−/−^* and *MPO^+/+^* mice were injected into the tail vein of recipient *LDLR^−/−^* mice. One *LDLR^−/−/^MPO^+/+tp^* mouse did not survive after bone marrow transplantation. After 10 weeks recovery, NASH was induced by feeding the mice a diet containing 17% casein, 0.3% DL-methionine, 34% sucrose, 14.5% cornstarch, 0.2% cholesterol, 5% cellulose, and 21% butter for 8 weeks (Scientific Animal Food and Engineering, Villemoisson-sur-orge, France) [12]. The engraftment efficiency was determined as previously described [14] and found to be 95.2%.To evaluate the effect of the intervention in relation to the diet key parameters assessed in the high-fat fed mice in the current study are compared with those obtained from chow-fed mice in a recently published parallel experiment [15].

### Tissue Specimens

Mice were sacrificed by CO_2_ asphyxation followed by removal of liver and mesenteric adipose tissue. Tissues were divided into pieces and 1) snap-frozen in liquid nitrogen for RT-PCR, ELISA, and lipid analysis, 2) fixed with formalin and embedded in paraffin for histopathology and immunohistochemistry, 3) snap-frozen in 2-methylbutane after embedding in Tissue-Tek OCT (Sakura Finetek, Zoeterwoude, the Netherlands).

### Lipid Analysis

Tail vein blood was collected after 4 hours fasting in heparin coated glass capillaries. Plasma and liver triglyceride and cholesterol were measured using the GPO-PAP kit according to the manufacturer’s instructions (Roche, Basel, Switzerland) after lipid extraction was performed using a modified Folch technique [16]. Protein content was measured by the BCA method (Pierce, Rockford, IL).

### Histology and Immunohistochemistry

Paraffin-embedded sections were cut and stained with haematoxylin and eosin for histopathological analysis and with Sirius red to study collagen distribution. The degree of steatosis, lobular inflammation, hepatocyte ballooning, and fibrosis was scored semi-quantitatively on a 3-point scale by an experienced animal pathologist. Frozen liver sections were immersed in Oil Red O/isopropanol (Sigma-Aldrich, Zwijndrecht, the Netherlands) to stain neutral lipids. Immunohistochemical staining for MPO, Ly-6G, Mac-1, F4/80, and CD3 was performed as previously described [5], [12], [14]. For quantification, six 200× fields were counted in a blinded fashion by two observers, and cell number was expressed relative to the sectioned area per mm^2^. MPO foci were defined as aggregation of >2 MPO-positive cells.

### Quantitative Real-time PCR

Total RNA isolation, reverse transcription, and real-time PCR was performed as previously described [5], using the primer sets presented in Table 1. Relative gene expression was normalised against cyclophilin A and β-actin gene expression.

**Table 1:** Primer sequences for quantitative RT-PCR.

Gene		Sequence
β-actin	Forward	GACAGGATGCAGAAGGAGATTACTG
	Reverse	CCACCGATCCACACAGAGTACTT
Cyclophilin A	Forward	TTCCTCCTTTCACAGAATTATTCCA
	Reverse	CCGCCAGTGCCATTATGG
TNF-α	Forward	CATCTTCTCAAAATTCGAGTGACAA
	Reverse	TGGGAGTAGACAAGGTACAACCC
IL-6	Forward	TTCAACCAAGAGGTAAAAGATTTACATAA
	Reverse	CACTCCTTCTGTGACTCCAGCTT
Mcp-1	Forward	GCTGGAGAGCTACAAGAGGATCA
	Reverse	ACAGACCTCTCTCTTGAGCTTGGT
Mac-1	Forward	ACTTTCAGAAGATGAAGGAGTTTGTCT
	Reverse	TGTGATCTTGGGCTAGGGTTTC
Adiponectin	Forward	AAGGAGATGCAGGTCTTCTTGGT
	Reverse	CCCCGTGGCCCTTCAG
Leptin	Forward	CACACACGCAGTCGGTATCC
	Reverse	GTCCATCTTGGACAAACTCAGAATG
SREBP1	Forward	GATGTGCGAACTGGACACAG
	Reverse	CATAGGGGGCGTCAAACAG
SREBF2	Forward	GCAGCAACGGGACCATTCT
	Reverse	CCCCATGACTAAGTCCTTCAACT
HMGCR	Forward	AGCTTGCCCGAATTGTATGTG
	Reverse	TCTGTTGTGAACCATGTGACTTC
SR-B1	Forward	TTTGGAGTGGTAGTAAAAAGGGC
	Reverse	TGACATCAGGGACTCAGAGTAG
CD36	Forward	ATGGGCTGTGATCGGAACTG
	Reverse	GTCTTCCCAATAAGCATGTCTCC
SR-A	Forward	CATACAGAAACACTGCATGTCAGAGT
	Reverse	TTCTGCTGATACTTTGTACACACGTT
BAMBI	Forward	GATCGCCACTCCAGCTACTTC
	Reverse	GCAGGCACTAAGCTCAGACTT
CD68	Forward	TGACCTGCTCTCTCTAAGGCTACA
	Reverse	TCACGGTTGCAAGAGAAACATG
ASMA	Forward	ACGAACGCTTCCGCTGC
	Reverse	GATGCCCGCTGACTCCAT
TGF-b1	Forward	GCCCTTCCTGCTCCTCATG
	Reverse	CCGCACACAGCAGTTCTTCTC
MMP-13	Forward	ACAAAGATTATCCCCGCCTCATA
	Reverse	CACAATGCGATTACTCCAGATACTG
Col1A1	Forward	AACCCTGCCCGCACATG
	Reverse	CAGACGGCTGAGTAGGGAACA
IL-1α	Forward	GCACCTTACACCTACCAGAGT
	Reverse	AAACTTCTGCCTGACGAGCTT
PAI-1	Forward	TGGATGCTGAACTCATCAGACAA
	Reverse	GCCAGGGTTGCACTAAACATG
TIMP1	Forward	GCAACTCGGACCTGGTCATAA
	Reverse	CGGCCCGTGATGAGAAACT

### Nitrotyrosine, Myeloperoxidase, and Alanine Amino Transferase ELISA

Liver samples were homogenized with a mini-bead beater and glass beads in lysis buffer (300 mM NaCl, 30 mM Tris-HCl (pH 7.4), 2 mM MgCl_2_, 2 mM CaCl_2_, 1% Triton X-100, in the presence of Pepstatin A, Leupeptin, and Aprotinin (all at 20 ng/ml)). Plasma and liver MPO and liver nitrotyrosine were measured using sandwich ELISA according to the manufacturer’s protocol (Hycult Biotechnology, Uden, the Netherlands). Plasma alanine amino transferase (ALT) was determined by ELISA (Antibodies-online, Aachen, Germany). Samples were analysed in duplicate in the same run. The intra-assay coefficient of variance was <10%.

### Hydroxyproline Assay

Hydroxyproline content of proteins was measured after acid hydrolysis with 6M HCl. Amino acid analysis was performed as recently described [17]. Briefly, samples were introduced into a tandem mass spectrometer using UPLC. Amino acids were measured in multiple reaction mode in ESI-positive mode. The mass transition 131.75>85.9 was used for the identification of hydroxyproline. Stable isotope-labelled asparagine was used as internal standard.

### Statistics

Data are represented as mean±SEM. Differences between groups were analysed using the Mann Whitney test, or one-way ANOVA with Dunnett’s test for multiple comparison. Statistical analyses were performed using Graphpad Prism 5.02 for Windows (Graphpad Software, San Diego, CA). A p value<0.05 was considered statistically significant.

## Results

### Hepatic MPO Accumulation in LDLR^−/−^ Mice after High-fat Feeding

**Figure 1:**
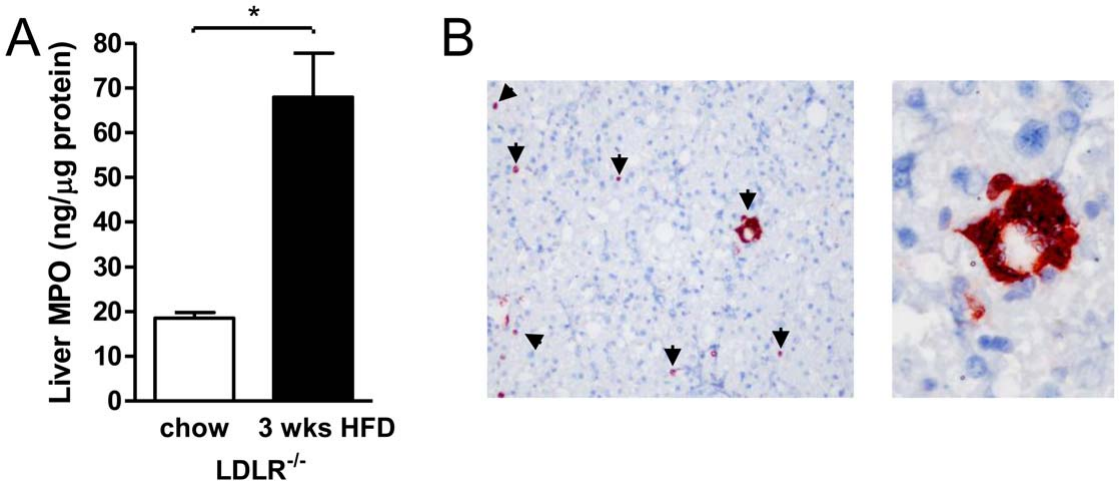
Strong high-fat diet-induced induction of MPO in the liver. A) Total liver MPO content of *LDLR* ^−/−^ mice as assessed by ELISA was almost four-fold increased by three weeks of high-fat feeding (19±1 vs. 68±10 ng/µg protein, p<0.01; n = 6 in both groups). B) MPO immunostaining reveals infiltration of neutrophils into the liver of *LDLR* ^−/−^ mice after three weeks of high-fat feeding (see arrows; 100× magnification). Many neutrophils are organized into aggregates predominantly surrounding steatotic hepatocytes (right panel; 200× magnification). MPO was not detected in Kupffer cells.

Hyperlipidemic mice such as *LDLR* ^−/−^ mice provide an excellent model for the study of NASH since they uniformly exhibit all of its phenotypic aspects, including hepatic inflammation and fibrosis, without requiring non-physiological diets [12], [14], [18]. Moreover, they exhibit insulin resistance [19], enabling mechanistic studies of NASH in the appropriate context of metabolic aberrations as observed in humans. Previously, high-fat feeding of these hyperlipidemic mice was shown to lead to elevated plasma MPO levels [12], [20]. We now assessed whether a three weeks high-fat diet also affected liver MPO, using previously described liver samples [12]. High-fat feeding caused a 3.7-fold increase of liver MPO protein in LDLR-deficient animals (p<0.01; Fig. 1a). This was associated with substantial infiltration of MPO-positive neutrophils into the liver, and not caused by expression of MPO in Kupffer cells (Fig. 1b). These neutrophils frequently assembled into aggregate structures around hepatocytes with macrovesicular steatosis, resembling the crown-like macrophage structures found in adipose tissue of obese animals [21]. Thus, hepatic lipid accumulation triggered by a high-fat diet is associated with liver neutrophil infiltration, leading to increased hepatic MPO protein levels.

### Reduced MPO Activity in LDLR^−/−/^MPO^−/−tp^ Mice

The accumulation of MPO in the liver upon high-fat feeding suggested that MPO might contribute to the pathogenesis of NASH. To further examine the role of MPO in NASH, we took advantage of the fact that MPO is exclusively expressed in the hematopoietic system [22], and performed bone marrow transplantation experiments that resulted in the generation of combined MPO-deficient, LDL-R-deficient mice (*LDLR* ^−/−/^ *MPO* ^−/−tp^) mice. *LDLR^−/−/^MPO^+/+tp^* controls and *LDLR^−/−/^MPO^−/−tp^* mice had a similar body weight, and diet-induced weight gain was comparable (*LDLR^−/−/^MPO^+/+tp^* from 19.9±0.4 to 21.8±0.3 g, *LDLR^−/−/^MPO^−/−tp^*: from 20.0±0.2 to 21.9±0.7 g).

**Figure 2:**
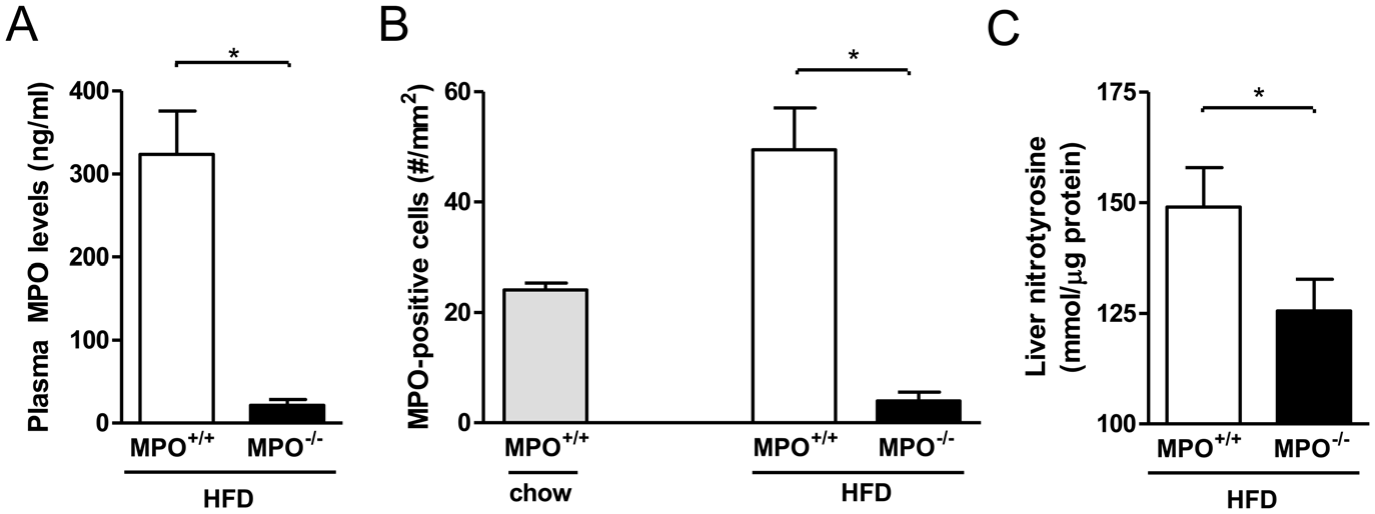
Reduced hepatic MPO and MPO-derived nitrated proteins in *LDLR* ^−/−/^ *MPO* ^−/−tp^ mice after 8 weeks of high-fat feeding. A) Plasma MPO levels of *LDLR* ^−/−/^ *MPO* ^−/−tp^ and *LDLR* ^−/−/^ *MPO* ^+/+tp^ mice (22±7 vs. 324±52 ng/ml, p<0.01). B) The MPO-positive cell number is strongly reduced in the liver of *LDLR* ^−/−/^ *MPO* ^−/−tp^ vs. *LDLR* ^−/−/^ *MPO* ^+/+tp^ mice (49.5±7.6 vs. 4.0±1.6 cells/mm^2^, p<0.01), and much lower than those observed in *LDLR* ^−/−/^ *MPO* ^+/+^ mice on chow. C) Hepatic levels of nitrotyrosine, a marker of MPO activity, are reduced in *LDLR* ^−/−/^ *MPO* ^−/−tp^ animals in comparison with *LDLR* ^−/−/^ *MPO* ^+/+tp^ mice (126±7 vs. 149±9 mmol/µg protein, p = 0.02).

After high-fat feeding, MPO plasma levels in the *LDLR* ^−/−/^ *MPO* ^+/+*tp*^ mice were 324 ng/ml whereas plasma from *LDLR* ^−/−/^ *MPO* ^−/−tp^ animals contained only 22 ng/ml of MPO (p<0.01; Fig. 2a). Similar to plasma MPO, liver MPO was very significantly reduced in the *LDLR* ^−/−/^ *MPO* ^−/−tp^ group after high-fat feeding as shown by quantification of MPO-positive cell numbers, with levels well under those observed in chow-fed *LDLR* ^−/−/^ *MPO* ^+/+^ mice(p<0.001; Fig. 2b).

To investigate if the marked reduction of hepatic MPO in *LDLR^−/−/^MPO^−/−tp^* mice translated into diminished generation of MPO-mediated cytotoxic products, we studied hepatic levels of nitrotyrosine, a protein modification generated at sites of inflammation as a result of the activity of several enzymes among which MPO, which accumulates in NAFLD [5], [8], [23]. As expected, mice in the *LDLR* ^−/−/^ *MPO* ^−/−tp^ group displayed significantly reduced levels of nitrotyrosine in the liver, consistent with reduced MPO activity (Fig. 2c; p<0.05).

### Characterization of NASH Severity

**Figure 3:**
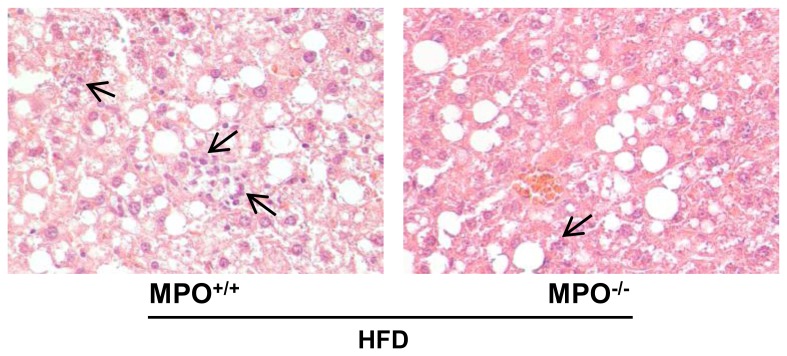
Liver histology of *LDLR* ^−/−/^ *MPO* ^−/−tp^ animals in comparison with *LDLR* ^−/−/^ *MPO* ^+/+tp^ mice after 8 weeks of high-fat feeding. Representative pictures of HE-stained liver sections of *LDLR* ^−/−/^ *MPO* ^−/−tp^ and *LDLR* ^−/−/^ *MPO* ^+/+tp^ mice indicating steatosis and inflammation (arrows).

Histological examination of the livers after H/E-staining revealed diffuse microvesicular and macrovesicular steatosis in both groups (Fig. 3). Semi-quantitative evaluation of the extent of steatosis showed no statistically significant differences between *LDLR* ^−/−/^ *MPO* ^+/+*tp*^ and *LDLR^−/−/^MPO^−/−tp^* mice (2.4±0.3 vs. 2.1±0.2, p = 0.43). Inflammatory cell foci were observed in both groups, but less frequently found in the *LDLR^−/−/^MPO^−/−tp^* group, although the difference was not statistically significant (0.75±0.31 vs. 0.50±0.27, p = 0.12). Hepatocyte ballooning, a feature of progressive human NASH, was not observed in either group. Plasma levels of ALT, a marker of hepatocyte injury, were lower in the *LDLR^−/−/^MPO^−/−tp^* mice (31.3±4.1 U/l vs. 43.2±3.8 U/l in *LDLR^−/−/^MPO^+/+tp^* mice, p<0.05).

### MPO Deficiency Leads to Reduced Liver Cholesterol but does not Affect Triglyceride Accumulation

**Figure 4:**
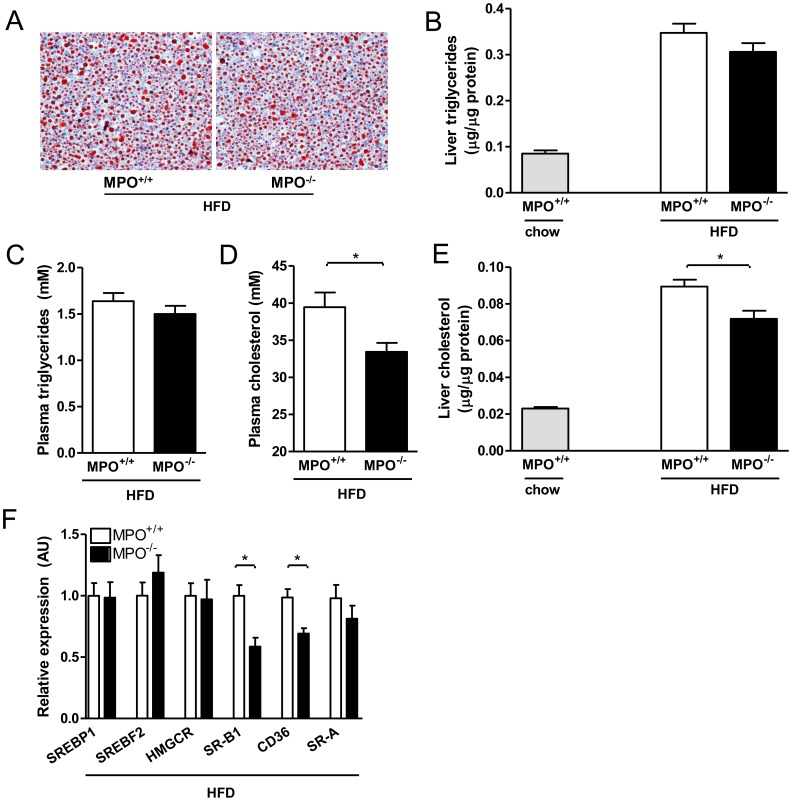
Decreased cholesterol accumulation in the liver of *LDLR* ^−/−/^ *MPO* ^−/−tp^ mice. A) Representative Oil red O stainings of liver sections of *LDLR* ^−/−/^ *MPO* ^−/−tp^ and *LDLR* ^−/−/^ *MPO* ^+/+tp^ mice fed a high-fat diet for 8 weeks, showing comparable lipid accumulation (100× magnification). B) Similar hepatic triglyceride levels in *LDLR* ^−/−/^ *MPO* ^−/−tp^ and *LDLR* ^−/−/^ *MPO* ^+/+tp^ mice after high-fat feeding (0.31±0.02 vs. 0.35±0.02 µg/µg protein, p = 0.24). Chow-fed *LDLR* ^−/−/^ *MPO* ^+/+^ mice show a lower level of liver triglycerides. C) Plasma triglyceride levels are similar in *LDLR* ^−/−/^ *MPO* ^−/−tp^ and *LDLR* ^−/−/^ *MPO* ^+/+tp^ animals after high-fat feeding (1.50±0.09 vs. 1.64±0.09 mmol/l, p = 0.42). D) High-fat feeding results in higher plasma cholesterol levels in *LDLR* ^−/−/^ *MPO* ^+/+tp^ animals as compared with *LDLR* ^−/−/^ *MPO* ^−/−tp^ mice (33.5±0.1 vs. 39.5±2.0 mmol/l, p = 0.02). E) Diet-induced liver cholesterol accumulation is reduced in *LDLR* ^−/−/^ *MPO* ^−/−tp^ mice compared with *LDLR* ^−/−/^ *MPO* ^+/+tp^ animals (0.072±0.004 vs. 0.090±0.004 µg/µg protein, p = 0.01), but does not reach the level observed in chow-fed *LDLR* ^−/−/^ *MPO* ^+/+^ mice. F) Hepatic mRNA expression of key enzymes in cholesterol metabolism is not altered in *LDLR* ^−/−/^ *MPO* ^−/−tp^ mice, whereas scavenger receptor expression is reduced (SR-B1 1.7-fold, p<0.01; CD36 1.4-fold, p<0.01, SR-A 1.2-fold, p = 0.63).

Next, the effects of MPO deficiency on the development of hepatic steatosis were examined in more detail. Oil red O staining of liver sections did not reveal obvious differences with respect to distribution or extent of lipid accumulation between *LDLR^−/−/^MPO^+/+tp^* mice and *LDLR^−/−/^MPO^−/−tp^* mice (Fig. 4a). In line with this, biochemical analysis revealed similar liver triglyceride content in *LDLR^−/−/^MPO^−/−tp^* and *LDLR^−/−/^MPO^+/+tp^* animals (p = 0.24; Fig. 4b). Plasma triglyceride levels were also similar in *LDLR^−/−/^MPO^+/+tp^* and *LDLR^−/−/^MPO^−/tp-^* mice (Fig. 4c). In contrast, both plasma and hepatic cholesterol levels were significantly lower in *LDLR^−/−/^MPO^−/−tp^* mice after the high-fat diet as compared with the *LDLR^−/−/^MPO^+/+tp^* animals (33.5±1.2 vs. 39.5±1.9 mM; p = 0.01, and 0.072±0.005 vs. 0.090±0.004 µg/µg protein; p = 0.01, respectively; Fig. 4d,e), though cholesterol accumulation in the *LDLR^−/−/^MPO^−/−tp^* group was still higher compared to *LDLR^−/−/^MPO^+/+^* animals on chow.

The differences in hepatic cholesterol do not appear to be due to altered synthesis, since hepatic gene expression of the two master regulators of cholesterol synthesis, SREBP1 and SREBF2, was comparable in both groups (p = 0.89, p = 0.32, respectively; Fig. 4f). Expression of hydroxymethylglutaryl-CoA reductase (HMGCR), the key rate-limiting enzyme in cholesterol synthesis, was also not significantly different (p = 0.42; Fig. 4f). Interestingly, however, expression of SR-A and CD36, two important proteins involved in the uptake of oxidized LDL, was lower in the *LDLR^−/−/^MPO^−/−tp^* group, although the difference was only significant for CD36 (p = 0.63, p<0.01, respectively; Fig. 4f). This may indicate reduced internalization of oxidized cholesterol in the liver of *LDLR^−/−/^MPO^−/−tp^* mice. Hepatic expression of SR-B1, a scavenger receptor mainly involved in the uptake of HDL-derived cholesterol and cholesteryl esters, was also decreased in *LDLR^−/−/^MPO^−/−tp^* mice (p<0.01, Fig. 4f).

### Generalized Attenuation of High-fat Diet-induced Liver Inflammation in LDLR^−/−/^MPO^−/−tp^ Mice

**Figure 5:**
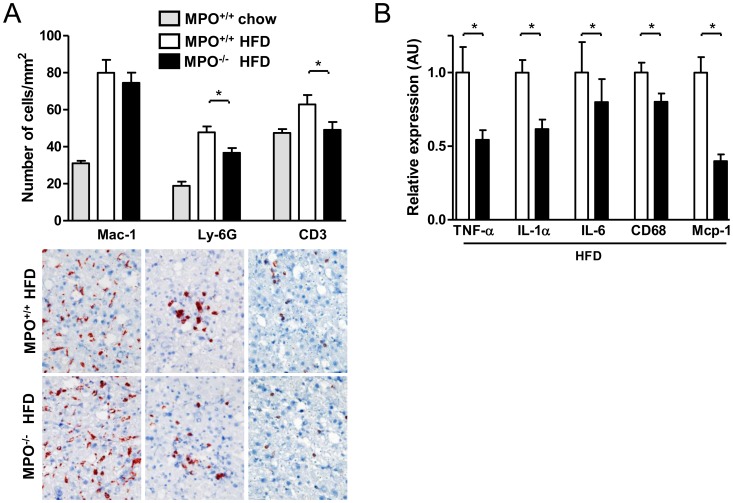
General reduction of diet-induced hepatic inflammation in *LDLR* ^−/−/^ *MPO* ^−/−tp^ mice. A) Significantly lower number of hepatic Ly-6G^+^ neutrophils and CD3^+^ T-lymphocytes in *LDLR* ^−/−/^ *MPO* ^−/−tp^ mice as compared with *LDLR* ^−/−/^ *MPO* ^+/+tp^ mice after 8 weeks of high-fat feeding (Ly-6G: 36.7±2.6 vs. 47.8±3.1 cells/mm^2^, p = 0.03; CD3: 49.1±4.2 vs. 62.9±5.0 cells/mm^2^, p = 0.04). Pictures represent examples of the stainings (200× magnification). B) Hepatic pro-inflammatory cytokine/chemokine expression is substantially reduced in *LDLR* ^−/−/^ *MPO* ^−/−tp^ mice after 8 weeks high-fat diet (TNF-α 1.8-fold, p = 0.03, IL-1α 1.6-fold, p<0.01, IL-6 1.3-fold, p = 0.67, Mcp-1 2.5-fold, p<0.01), in parallel with a reduction of CD68 expression (1.3-fold, p<0.05).

Active MPO has powerful pro-inflammatory effects, partly attributable to the generation of oxidized cholesterol [7], [24]. Therefore, we next investigated the effect of MPO deficiency on hepatic inflammation following high-fat feeding. The cellular nature of the inflammation was investigated by immunohistochemical analysis of Mac-1, Ly-6G, and CD3, markers of Kupffer cells/macrophages, neutrophils, and T-lymphocytes, respectively. Interestingly, the number of neutrophils and T-lymphocytes was significantly reduced in the liver of *LDLR^−/−/^MPO^−/−tp^* mice (p<0.05, p<0.05, respectively; Fig. 5a), and similar to the numbers observed in *LDLR^−/−/^MPO^+/+^* mice on chow. Moreover, additional analyses of *LDLR^−/−/^MPO^−/−tp^* animals consistently revealed a strongly reduced expression of pro-inflammatory genes previously implicated in the pathogenesis of NASH (Fig. 5b). For example, tumor necrosis factor-α (TNF-α) and IL-1α mRNA expression were almost two-fold lower in the liver of *LDLR^−/−/^MPO^−/−tp^* mice compared with *LDLR^−/−/^MPO^+/+tp^* mice (p<0.05, p<0.01, respectively). In addition, hepatic IL-6 expression tended to be reduced in *LDLR^−/−/^MPO^−/−tp^* mice relative to *LDLR^−/−/^MPO^+/+tp^* mice, although the difference was not statistically significant. Hepatic monocyte chemoattractant protein-1 (Mcp-1) mRNA expression was over two-fold lower in *LDLR^−/−/^MPO^−/−tp^* mice (*p*<0.01), and, consistent with this, their CD68 mRNA expression was also significantly reduced. Taken together, these results show that MPO plays an important role in high-fat diet-induced inflammation of the liver, promoting both inflammatory cell recruitment and cytokine/chemokine expression.

### Reduced High-fat Diet-induced Adipose Tissue Inflammation in LDLR^−/−/^MPO^−/−tp^ Mice

**Figure 6:**
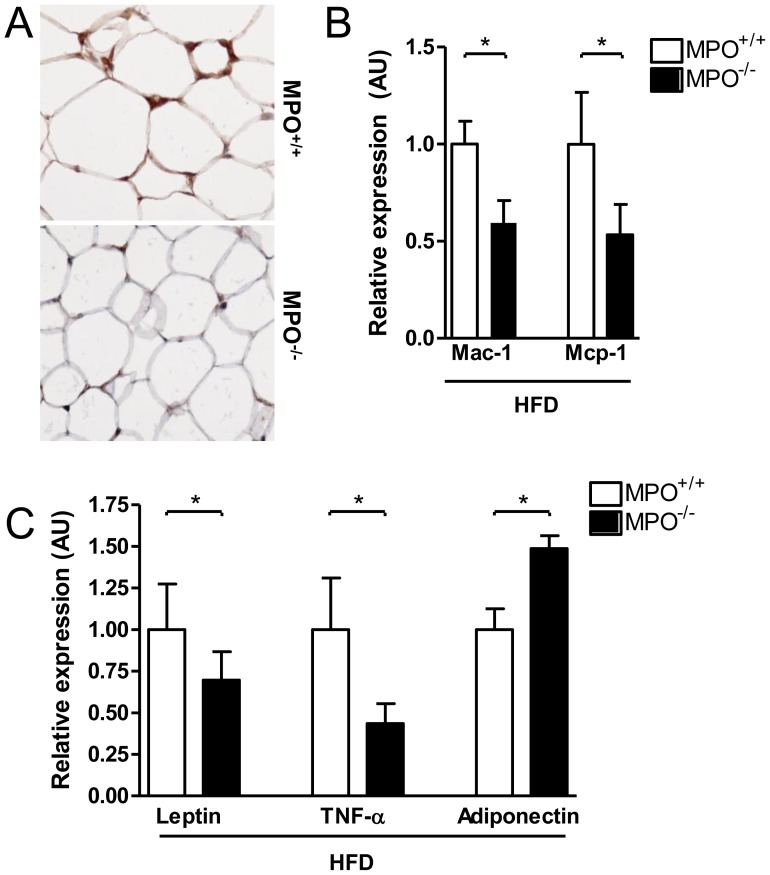
Reduced diet-induced adipose tissue inflammation in *LDLR* ^−/−/^ *MPO* ^−/−tp^ mice. A) Lack of high-fat diet-associated macrophage ‘crown-like structures’ in visceral adipose tissue of *LDLR* ^−/−/^ *MPO* ^−/−tp^ mice as revealed by F4/80 immunostaining (200× magnification). B) Adipose tissue mRNA expression of the macrophage marker Mac-1 and the macrophage chemokine Mcp-1 is significantly lower in *LDLR* ^−/−/^ *MPO* ^−/−tp^ mice fed a high-fat diet for 8 weeks (p<0.05). C) Reduced adipose tissue expression of the adipokines leptin and TNF-α in *LDLR* ^−/−/^ *MPO* ^−/−tp^ mice after 8 weeks of high-fat feeding, whereas expression of adiponectin is increased.

The pathogenesis of NASH is mediated by cross-talk between inflamed adipose tissue and the liver [25]. In order to investigate the potential contribution of adipose tissue-derived factors to the reduced hepatic inflammation in LDLR^−/−/^MPO^−/−tp^ mice, several inflammatory parameters were investigated. First of all, Ly-6G staining revealed an absence of neutrophils in visceral adipose tissue in both groups (data not shown). Next, visceral adipose tissue was stained for the macrophage marker F4/80. High-fat diet-induced obesity is characterized by infiltration of macrophages into adipose tissue, where they organize into so-called ‘crown-like structures’ surrounding dead adipocytes [21]. Interestingly, adipose tissue of LDLR^−/−/^MPO^−/−tp^ mice was completely devoid of such crown-like structures, whereas they were readily identifiable in adipose tissue of LDLR^−/−/^MPO^+/+tp^ mice (Fig. 6a). Quantitative PCR analysis of adipose tissue Mac-1 expression, another macrophage marker, was in line with these results, showing a marked reduction in LDLR^−/−/^MPO^−/−tp^ animals (p<0.05; Fig. 6b). Similarly, expression of Mcp-1, a potent chemo-attractant for monocytes, was strongly reduced in the LDLR^−/−/^MPO^−/−tp^ group (p<0.05; Fig. 6b). Furthermore, adipose tissue expression of the pro-inflammatory adipokines leptin and TNF-α was lower in LDLR^−/−/^MPO^−/−tp^ animals, whereas expression of adiponectin, which has anti-inflammatory properties, was higher (Fig. 6c). Thus, MPO deficiency protects adipose tissue from high-fat diet-induced inflammation, which may contribute to the attenuation of inflammation in the liver of LDLR^−/−/^MPO^−/−tp^ mice.

### Decreased Liver Fibrosis in LDLR^−/−/^MPO^−/−tp^ Mice

**Figure 7:**
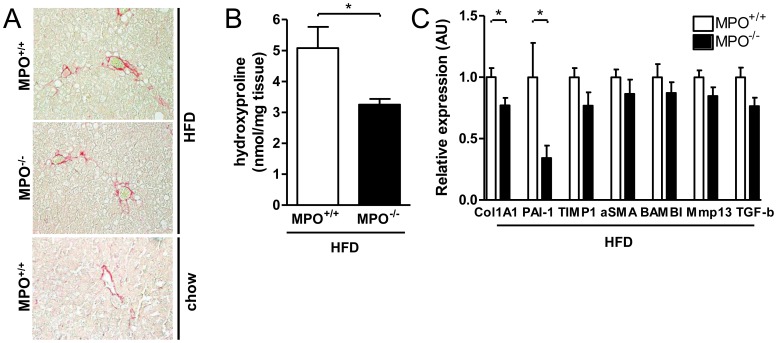
Attenuation of liver fibrosis in *LDLR* ^−/−/^ *MPO* ^−/−tp^ mice after 8 weeks high-fat diet. A) Sirius red staining of liver sections of *LDLR* ^−/−/^ *MPO* ^−/−tp^,*LDLR* ^−/−/^ *MPO* ^+/+tp^, and chow-fed *LDLR* ^−/−/^ *MPO* ^+/+^ mice (200× magnification). B) The amount of hydroxyproline, a protein modification specifically present in collagen and elastin, is significantly lower in the liver of *LDLR* ^−/−/^ *MPO* ^−/−tp^ mice in comparison with *LDLR* ^−/−/^ *MPO* ^+/+tp^ mice (3.3±0.2 vs. 4.6±0.7 nmol/mg tissue, p<0.01). C) *LDLR* ^−/−/^ *MPO* ^−/−tp^ mice display reduced expression of collagen type I, PAI-1, TIMP1, α-SMA, MMP-13, TGF-β1, and BAMBI, genes associated with hepatic fibrosis.

Progression of NAFLD, mediated by sustained inflammation, ultimately results in the development of hepatic fibrosis. Since MPO exerts strong effects on various mechanisms involved in fibrogenesis and has been implicated in pro-fibrotic states in various other chronic inflammatory conditions, we next evaluated parameters of fibrosis in the liver of *LDLR^−/−/^MPO^−/−tp^* and *LDLR^−/−/^MPO^+/+tp^* mice. As expected in this dietary model of NASH, Sirius red staining of collagen in liver sections indicated only mild fibrosis in both groups (Fig. 7a). However, collagen content appeared to be slightly decreased in *LDLR^−/−/^MPO^−/−tp^* as compared to *LDLR^−/−/^MPO^+/+tp^* mice. More detailed quantitative biochemical analysis of the collagen/elastin content in liver homogenates as determined by hydroxyproline quantity revealed a lower amount in *LDLR^−/−/^MPO^−/−tp^* mice (p<0.01), supporting that their liver was less fibrotic (Fig. 7b). This was further substantiated by the fact that hepatic gene expression of collagen 1A1 was lower in the *LDLR^−/−/^MPO^−/−tp^* group (p<0.05; Fig. 7c). Moreover, mRNA levels of PAI-1, an important regulator of hepatic fibrosis, were significantly reduced in these animals (p<0.01; Fig. 7c). Expression of other fibrosis-related parameters such as tissue inhibitor of metalloproteinase 1 (TIMP1), α-smooth muscle actin (α-SMA), MMP-13, TGF-β1, and BAMBI was also reduced although not to a statistically significant extent (p = 0.15, p = 0.19, p = 0.12, p = 0.06, p = 0.39, respectively); Fig. 7c). Overall, these data suggest that MPO may promote the progression of NAFLD towards more advanced stages with fibrosis.

## Discussion

Hepatic inflammation is one of the defining criteria in the diagnosis of NASH, and primarily characterized by the abundant presence of neutrophils [26]. Neutrophils are equipped with formidable enzyme systems that generate factors with a high potential of causing tissue damage, most prominently represented by MPO. The results of the present study point to an important role for MPO in the development of NASH by increasing hepatic cholesterol accumulation, inflammation, and fibrosis.

The effect of MPO deficiency on plasma lipid levels and inflammation was previously studied in the context of atherosclerosis [20], [27]. In line with our findings, plasma triglyceride levels were comparable between *LDLR^−/−/^MPO^−/−^* and *LDLR^−/−/^MPO^+/+^* mice, whereas plasma cholesterol was lower in mice lacking MPO. We now report that hepatic cholesterol levels are also reduced in *LDLR^−/−/^MPO^−/−tp^* mice after high-fat feeding. There are several mechanisms by which MPO might affect plasma and liver cholesterol levels. MPO is known to inhibit cholesterol efflux from lipid-laden macrophages by oxidizing apoA-I in HDL [28]. MPO is also able to oxidize other apolipoproteins including apoB, an important component of LDL [29]. This may alter interaction with hepatocyte receptors and contribute to dyslipidemia by affecting clearance. Furthermore, nitrotyrosine formation on apoB-100 leads to enhanced uptake of cholesterol-containing LDL by macrophages [30]. Identification of the relative importance of these mechanisms for the observed reduction of total liver and plasma cholesterol levels will require detailed analysis of lipoprotein profiles. In view of mounting evidence that Kupffer cells acquire characteristics similar to lipid-laden macrophages/foam cells in response to high-fat feeding [2], [12], reduced hepatic cholesterol in *LDLR^−/−/^MPO^−/−tp^* mice may be related to diminished formation of foamy Kupffer cells.

Regardless of the mechanism, the reduced cholesterol levels in *LDLR^−/−/^MPO^−/−tp^* animals are significant in light of recent data indicating that cholesterol plays a pivotal role in the induction of inflammation in NASH [12], [31]. In this context, scavenging of oxidized cholesterol/lipoprotein particles by Kupffer cells and hepatocytes may be an initiating factor. Uptake of oxidized LDL/HDL is mediated by scavenger receptors, and is associated with chronic inflammation [32]. Of note, scavenger receptor expression is regulated by oxidized LDL through a positive feedback loop [33]. The reduced expression of CD36 that we found in the liver of *LDLR^−/−/^MPO^−/−tp^* mice may therefore be indicative of lower oxidized LDL levels, consistent with lower MPO activity. Future studies on the levels of oxidized cholesterol in plasma and liver are required to further define the mechanisms involved.

In addition to the pro-inflammatory effects related to cholesterol accumulation and modification, MPO can promote inflammation in various other ways. Firstly, MPO-mediated generation of HOCl and NO_2_ radicals directly results in chlorination and nitration of proteins and nucleic acids [7] reflecting cellular damage, a potent inducer of inflammation. The fact that hepatic nitrotyrosine levels were lower in *LDLR^−/−/^MPO^−/−tp^* mice suggests that MPO-mediated protein nitration may indeed contribute to high-fat diet-induced hepatic inflammation. Secondly, MPO and MPO-derived HOCl activate NF-κB signalling and increase TNF-α production by macrophages and other leukocytes [9], [10], [34]. This is consistent with our observation of lower hepatic and adipose tissue TNF-α expression in the *LDLR^−/−/^MPO^−/−tp^* mice, and may be related to an interaction between neutrophil-derived MPO and hepatic macrophages. Importantly, pro-inflammatory TNF-α and NF-κB signalling are key factors in the progression of NAFLD [1]. Thirdly, MPO activity is linked to lipid peroxidation, which is a prominent characteristic of fatty livers, promoting activation of stellate cells and attraction of inflammatory cells [35], [36]. Indeed, the substantial reduction of hepatic neutrophils and T-lymphocytes in *LDLR^−/−/^MPO^−/−tp^* mice provides supporting evidence for an important role of MPO-mediated lipid peroxidation in chemo-attraction of leukocytes in NASH.

Interestingly, we observed reduced numbers of adipose tissue macrophages in *LDLR^−/−/^MPO^−/−tp^* mice. This is in line with recent data indicating that high-fat diet-induced infiltration of macrophages into adipose tissue is preceded by neutrophil infiltration [37]. Moreover, lipid peroxidation is known to be markedly elevated in adipose tissue of obese mice [38]. Hence, our findings suggest that the reported early diet-induced sequestration of neutrophils in adipose tissue may promote lipid peroxidation via MPO-dependent mechanisms. Furthermore, accumulation of oxidized lipids in adipose tissue is associated with dysregulated adipokine expression [38], which is in line with our data on leptin and adiponectin expression. Importantly, reduced adiponectin and increased leptin secretion by adipose tissue promotes lipid accumulation, inflammation, and fibrogenesis in the liver [39].

Next to dysregulated adiponectin and leptin expression, numerous other factors modulated by MPO and MPO-derived products affect the development of fibrosis. For example, MPO-generated oxidants activate matrix metalloproteinases [40] while inhibiting protease inhibitors such as TIMP1 [41]. These actions are thought to suppress fibrosis. In contrast, high levels of MPO-derived HOCl can also inactivate matrix metalloproteinase 7 [42], thereby promoting fibrosis. Furthermore, MPO-related lipid peroxidation products stimulate stellate cell synthesis of type I collagen, the major collagen of the fibrotic liver [43], which expression was significantly reduced in the *LDLR^−/−/^MPO^−/−tp^* mice. Finally, HOCl fragments the extracellular matrix [11], which is associated with stellate cell activation as well. Our data indicate that *in vivo*, the pro-fibrotic effects of MPO may outweigh anti-fibrotic processes in the context of NASH, even though the fibrosis we observed was still very mild.

Our findings are likely to be clinically important since human NAFLD is associated with high numbers of MPO-expressing cells and accumulation of HOCl-modified and nitrated proteins [4], [5], [8]. Furthermore, there is strong evidence for increased oxidative stress and extensive lipid peroxidation in human NASH [4], [36], [44]. In this regard it is also important to note that in comparison to the mouse, human blood contains 5–7 times more neutrophils with a longer half-life, each containing about 10-fold more MPO [6], [45]. As such, it is likely that the contribution of MPO to the progression of NAFLD in man is more pronounced. Moreover, high and sustained MPO activity results in oxidative DNA damage [46], which is associated with the ultimate and most devastating complication of NASH, hepatocellular carcinoma [36].

In conclusion, we have shown that MPO-deficiency diminishes high-fat diet-induced NASH by reducing hepatic cholesterol accumulation, inflammation, and fibrosis. Furthermore, our data indicate a general role for MPO in the chronic inflammation associated with obesity and insulin resistance, and therefore argue for a re-evaluation of the role of neutrophils and their cytotoxic products in the pathogenesis of metabolic disease.
